# Bacteria [bak-tēr′-ē-ә]

**DOI:** 10.3201/eid3206.251514

**Published:** 2026-06

**Authors:** Hellen C.O. Santos-Dutra, Caroline C.P. da Costa, Diogo Nery Maciel, Rodrigo S. Santos, Mônica S. Barbosa

**Affiliations:** Federal University of Goiás, Goiânia, Goiás, Brazil

**Keywords:** bacteria, pathogens, etymology

A bacterium is a unicellular prokaryotic microorganism, and bacteria is the plural form that refers to these organisms as a group, historically classified within the former Monera kingdom (Figure). The term bacteria derives from Greek *baktērion* (βακτήριον), meaning small staff or cane, describing rod-like forms observed in early microscopic studies. Christian Gottfried Ehrenberg introduced the term bacterium in 1838, before modern bacteriology, and bacteria later became the plural form. Once viewed as microscopic plants, bacteria were later recognized as a distinct group of prokaryotic life and are now essential in ecologic processes, biotechnology, and public health, as beneficial or pathogenic agents.

**Figure:**
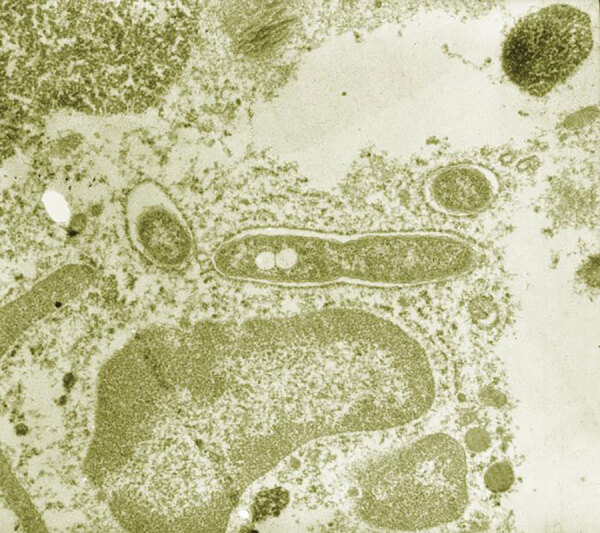
Ultrastructural morphology of numerous *Legionella pneumophila *bacteria in tissue section. Image from https://phil.cdc.gov/
